# Rigorous Statistical Methods for Rigorous Microbiome Science

**DOI:** 10.1128/mSystems.00117-19

**Published:** 2019-05-28

**Authors:** Amy D. Willis

**Affiliations:** a Department of Biostatistics, University of Washington, Seattle, Washington, USA

**Keywords:** hypothesis testing, machine learning, modeling, reproducibility, statistics

## Abstract

High-throughput sequencing has facilitated discovery in microbiome science, but distinguishing true discoveries from spurious signals can be challenging. The Statistical Diversity Lab develops rigorous statistical methods and statistical software for the analysis of microbiome and biodiversity data. Developing statistical methods that produce valid *P* values requires thoughtful modeling and careful validation, but careful statistical analysis reduces the risk of false discoveries and increases scientific understanding.

## Abstract (precis)

High-throughput sequencing has facilitated discovery in microbiome science, but distinguishing true discoveries from spurious signals can be challenging. The Statistical Diversity Lab develops rigorous statistical methods and statistical software for the analysis of microbiome and biodiversity data.

## PERSPECTIVE

The ever-increasing amount of data about microbial communities should be leading to an increasing understanding of the role of the microbiome in human and environmental health. However, the increasing amount and dimension of microbiome data may be drowning us in “significant” but irreproducible findings. Statistical models are a natural framework for dealing with uncertainty and variation and, when used correctly, have the potential to distinguish spurious signals from strong evidence (1–3). Unfortunately, it is substantially more difficult to develop a valid statistical hypothesis test than it is to develop a procedure that produces *P* values. My research group develops rigorous statistical methods, including valid and powerful hypothesis tests, and predictive models with high (but not overstated) accuracy and precision. Correctly identifying the microbial features that discriminate healthy and diseased microbiomes allows the scientific community to pursue promising avenues of research without becoming sidetracked by spurious correlations.

## WHY BOTHER WITH RIGOROUS STATISTICS?

To illustrate my philosophy of statistics, I want you to think about the last microbiome investigation that you performed (or read about). If you were able to repeat your experiment infinitely many times and with perfect precision, you could perform any quantitative analysis you wish and know that you got the correct answer. You could know if a particular gene in a particular genome conferred disease risk, or the average effect of the concentration of a strain on a functional trait of the environment. Statisticians call this type of data a census: perfect sampling of the entire population. Of course, we are never able to collect a microbial census, and our precision is limited by our measurement tools, such as sequencing machines. My approach to microbiome statistics involves using your finite amount of imprecise data to estimate the parameter that you would care about if you had infinite data and perfect precision. Critically, my approach always involves estimating the uncertainty in the parameter estimate, or the expected error rate of a prediction. By combining estimates with uncertainties, we can understand the strength of collected data to support a hypothesis.

Unfortunately, microbiologists frequently analyze their data as if they were census data (4, 5). Census data (to first approximation) do not require statistical modeling: any summary of the data is correct. An example of analyzing microbiome data as if they were census data is the analysis of Shannon diversity, which measures community evenness and richness. Plugging in the observed relative abundances to calculate Shannon diversity neglects to correct for bias due to undersampling, and microbiologists typically do not report error bars on their estimates of Shannon diversity (6). We developed a method to estimate *α*- and *β*-diversity and their uncertainties in reference 7. The example of Shannon diversity highlights that there are common microbiome data analyses that do not consider that the data are incomplete. Other common statistical errors in microbiome data analysis include *P* value hacking, not understanding the assumptions and limitations of the data analyses employed, not investigating the robustness of findings to preprocessing parameters, and performing statistical inference on a data-driven subset of parameters (e.g., doing statistical inference or quoting *P* values only for genes that have the largest observed effect size).

## WHAT IS A VALID HYPOTHESIS TEST?

A valid hypothesis test produces correct *P* values. *P* values are ubiquitous in the microbiome literature for testing the hypothesis that there is a null result. A standard hypothesis test evaluates if the data conflict with the null hypothesis. If they do, we reject the null hypothesis in favor of the alternative that there is a nonnull result. This parallels our legal system: innocent (null) until proven guilty (not null). We don’t declare innocence, we declare insufficient evidence of guilt.

A hypothesis test is valid if it incorrectly rejects the null hypothesis with a prespecified probability (e.g., 5%). *P* values measure the strength of evidence against the null: small *P* values suggest strong evidence against the null. A *P* value of 0.01 indicates that if the null hypothesis were true, what we observed (or an even stronger result) had less than a 1% chance of occurring. One percent isn’t very likely, so we reject the null hypothesis.

Unfortunately, there are many procedures that appear to be valid hypothesis tests but are not. I call these *P* value-generating procedures. A *P* value-generating procedure is any method that is not a valid hypothesis test but produces a number that it calls a *P* value.

When writing a microbiome paper, you may have to choose between different options for producing *P* values. Imagine you are interested in understanding the gene-level differences between one type of microbiome and another. You would typically collect some data (for example, using shotgun sequencing) and then decide on a method to test the hypothesis of no difference between the two types of microbiomes. Suppose one option, method 1, returns *P < *0.0001 and method 2 returns *P = *0.19. Which would you use in your paper?

Method 1 produces a result that is appealing (it appears you made a discovery!), but is method 1 a valid hypothesis test? If method 1 always returned *P < *0.0001, regardless of the input data, it would not be a valid hypothesis test. This is because it always rejects the null hypothesis, even when it is true.

Method 2 may be frustrating, but it may save you from publishing a false result. However, method 2 may just have randomly generated a number between 0 and 1. If every number between 0 and 1 had equal probability of being chosen, method 2 would be a valid hypothesis test—but not a good one. It has low power, or a low probability of correctly concluding that there is a difference between the communities (if we reject when *P < *0.05, its power is 5%). In comparison, method 1 appears to have extremely high power. However, it is not a valid hypothesis test, so that power is meaningless.

## HOW CAN WE IMPROVE THE POWER AND ACCURACY OF STATISTICAL METHODS?

While the random *P* value of method 2 is a valid hypothesis test, it is not one that we use because it has low power. An obvious way to improve the power of a hypothesis test is to use the data that you collected. For example, “corncob” uses amplicon sequencing data to test hypotheses about the relative abundance of microbes (8). “betta” tests the hypothesis that the diversity (either genetic or taxonomic) of a community is unchanged (4). These hypothesis tests are more complicated than methods 1 and 2, but they are valid (unlike method 1) and have high power (compared to method 2).

As more and more microbiology papers are published, and the excitement surrounding microbiome science grows, researchers will need rigorous statistical methods to decide which of many seemingly significant signals to pursue and which to ignore. In the absence of rigorous statistical methods, invalid hypothesis tests (like method 1) become popular. I hope that the demand for more rigorous approaches increases before microbiome science finds itself in a “reproducibility crisis” akin to that of cancer biology (9).

I anticipate that major advances will come from incorporating more data structures into statistical methods, and the Statistical Diversity Lab is pursuing methods development in this direction. The recent success of computational methods that use negative controls to detect and remove contamination (e.g., “decontam” [10]) leads me to predict that statistical methods that take advantage of data structures like dilution series, positive controls, and spike-ins will improve our power to make interesting but true discoveries. A similarly promising avenue is using existing data in conjunction with new data or searching for discoveries using multiple cohorts (11, 12). Barriers that need to be overcome before this can be realized include the development and validation of methods that remove study-specific variation (13, 14). Other research groups that develop, document, and use rigorous statistical and computational methods for microbiome data include the Statistical Genetics and Genomics Laboratory (U Penn), the Holmes Lab (Stanford), the Callahan Lab (NCSU), and the Fukuyama Lab (Indiana U).

Hypothesis testing is not the only way to use data to learn about microbiomes. Accurately predicting the response of a microbial community to a treatment is arguably more interesting than finding significant correlations. Accurate predictions require ample training data, which necessitates using data from distributed collaborations. Statistical and computational methods that calibrate data collected from different sources to remove study- or protocol-specific artifacts will be critical to detecting biological features rather than artifactual features (15).

## CONCLUSION

The coming years will see great advances in the technology that is available to answer important microbiome questions, and new computational methods will be developed to leverage the new data. However, we will need to develop, value, and use valid statistical methods to sort true discoveries from the noise. More than 85% of 1,500 surveyed scientists agreed that a better understanding of statistics would be likely or very likely to improve reproducibility (9), and I am working to provide methodological and educational resources for the microbiome community. By developing rigorous statistical methods, the Statistical Diversity Lab helps you determine if your discovery is signal or noise.
